# Monocyte/lymphocyte ratio predicts the severity of coronary artery disease: a syntax score assessment

**DOI:** 10.1186/s12872-017-0507-4

**Published:** 2017-03-31

**Authors:** Hanhua Ji, Yang Li, Zeyuan Fan, Bo Zuo, Xinwen Jian, Li Li, Tao Liu

**Affiliations:** 1 Department of Cardiology, Civil Aviation General Hospital, Civil Aviation Clinical Medical College of Peking University, No.1, Gaojingjia, Chaoyang District, Beijing, 100100 China; 2 Department of Cardiology, Peking University Third Hospital, Beijing, 100100 China

**Keywords:** Monocyte to lymphocyte ratio, Coronary artery disease, Syntax score

## Abstract

**Background:**

We aimed to explore whether monocyte to lymphocyte ratio (MLR) provides predictive value of the lesion severity in patients with coronary artery disease (CAD).

**Methods:**

Five hundred forty-three patients undergoing coronary angiography were analyzed in this retrospective study. Patients with coronary stenosis were divided into three groups on the basis of Syntax score. The control group consisted of patients with normal coronary arteries. MLR was calculated by dividing monocytes count by lymphocytes count obtained from routine blood examination. Multivariate logistic analysis was used to assess risk factors of CAD. Ordinal logistic regression analysis was used to assess the relationship between MLR and the lesion severity of coronary arteries.

**Results:**

MLR was found to be an independent risk factor of the presence of CAD (OR: 3.94, 95% CI: 1.20–12.95) and a predictor of the lesion severity (OR: 2.05, 95% CI: 1.15–3.66). Besides, MLR was positively correlated with Syntax score(*r* = 0.437, *p* < 0.001). In the receiver-operating characteristic (ROC) curve analysis, MLR, with an optimal cut-off value of 0.25, predicted the severe coronary lesion with a sensitivity of 60.26% and specificity of 78.49%.

**Conclusions:**

MLR was an independent risk factor of the presence of CAD, and a predictor of the lesion severity. Compared to neutrophil to lymphocyte ratio (NLR), MLR has better performance to reflect the severity of coronary lesion.

## Background

CAD is still a major contributor of mortality in patients with cardiovascular diseases, although much effort has been made to improve disease management including intensive medical care and invasive procedures over the last decade [1–5]. The severity of coronary lesion is still a crucial factor in the stratification of cardiovascular risk to determine the optimal treatment strategy. Several randomized Syntax trials have released the results that Syntax Score system, could be applied as a grading method to determine risk level, taking into account: number of disease segments, tortuosity, calcification, presence of thrombus, lesion length, dominance, bifurcation, trifurcation, aorto-ostial lesions, diffuse disease, and total occlusion, and each coronary lesion with a diameter stenosis ≥ 50% in vessels ≥ 1.5 mm was scored [6–8].

As a major contributor on the initiation and aggravation of atherosclerosis, inflammation, regulated by immune cells, could accelerate atherosclerotic progression, ultimately causing plaque rupture and serious adverse events related to CAD [9, 10]. The existence of inflammatory cells, such as monocytes, macrophages, dendritic cells and T cells, is a common characteristic in formation of atherosclerotic lesions [11–13]. In addition, immune cells, cytokines and other biomedical markers implicated in inflammatory response have been investigated to predict progression/severity of the lesion and explore pathological mechanism of the arterial disease [14].

Increased values of white blood cells and subtypes, such as eosinophils, monocytes, neutrophils, and lymphocytes, have been found to be closely related with the cardiovascular adverse events [15, 16]. NLR and MLR are novel indicators of baseline inflammatory response. NLR has been reported to be an independent factor of clinical outcomes in coronary artery disease, and an attractive biomarker for predicting the severity of the lesion, which has potential to be a universal biomarker in clinical applications [17–20]. MLR has been proved to be a prognostic factor in patients with malignancies and tuberculosis [21–24]. However, the role of MLR in CAD is still unknown. Therefore, we designed this study to investigate whether MLR is an independent risk factor of CAD, and could be useful for patient risk-stratify. To the best of our knowledge, this is the first report on the relationship between MLR and CAD.

## Methods

### Study population

Five hundred forty-three participants who underwent coronary angiography for suspected or known coronary atherosclerosis at the Civil Aviation General Hospital were retrospectively analyzed in the project between January 2014 and August 2015. The study protocol was approved by the Ethical Committee of Civil Aviation General Hospital, and informed consent was obtained from all patients enrolled. Exclusion criteria were congenital heart disease, valvular heart disease, severe heart failure, hematonosis, liver or renal dysfunction, stroke, tumor, thyroid disease, autoimmune disease and infectious diseases. Enrolled patients underwent clinical investigation for systematic evaluation of cardiac function and were requested for the information of family history of CAD and history of smoking, previous CAD, hypertension, diabetes and non-heart diseases. The diagnosis of CAD was based on current AHA/ACC guidelines [25]. Hypertension was defined as systolic blood pressured ≥140 mmHg and/or a diastolic blood pressure ≥ 90 mmHg taken from at least two-times, or current use of an antihypertensive medication. Diabetes mellitus was defined as fasting plasma glucose level ≥7.0 mmol/l or casual plasma glucose level ≥ 11.1 mmol/l or active use of an antidiabetic agent.

### Laboratory analysis

Venous blood samples of all patients were drawn from upper limb. Patients were advised to fast at least for 12 h before blood investigations. The blood routine and biochemical indicators were measured by clinical laboratory of our hospital. The biochemical indicators included fasting blood-glucose (FBG), total cholesterol (TC), triglycerides (TG), low density lipoprotein cholesterol (LDL), high density lipoprotein cholesterol (HDL), serum creatinine (SCr) and uric acid (UA). The biochemical indicators were determined by an automatic biochemical analyzer (Beckman Coulter, CA, USA).

### Assessment of the severity of CAD

Coronary angiogram was assessed by two interventional physicians blindly. The CAD was defined as the existence of significant narrowing (≥50%) in any of the main coronary arteries, according to coronary artery lesion classification of AHA/ACC. Coronary artery of the control group in the study was defined by the presence of < 50% stenoses.

Diagnostic angiograms were scored based on the Syntax Score. Syntax score is a relatively mature evaluation system to reflect the coronary stenosis prospectively. Georgios et al have reported the full details on Syntax score calculation [7]. Additionally, CAD patients were divided into three groups based on the Syntax score (mild =1–22, moderate 23–32, and severe ≥ 33).

### Statistical analysis

Continuous variables were defined as mean ± SD or median (interquartile range); categorical variables were expressed as percentages. For continuous variables, the Kolmogorov-Smirnov test was applied to test the normality of distribution, *t* test or the Mann-Whitney *U* test, one-way ANOVA model was used to compare. For categorical variables, the chi-square test was used. Spearman rank test was used to test correlations. ROC curve analysis was performed to verify the diagnostic accuracy of MLR level in the presence and severity of CAD. Binary and ordinal logistic regression analysis was used to assess the independent predictors of CAD and coronary lesion severity respectively. Statistical analyses were performed using SPSS 15.0. A statistically significance was taken as a 2-tailed *p* < 0.05.

## Results

### Baseline characteristics of the study population

Baseline demographic and biochemical characteristics of all 543 patients were outlined in Table 1. Study subjects consisted of 381 patients with CAD (CAD group, 55% male: age 63 ± 10 years) and 162 patients with normal artery conditions (control group, 35% male: age 55 ± 9 years). Patients with CAD were a bit older, and had more conventional CAD risk factors. The level of fasting blood glucose and creatinine were higher in CAD group. Compared to the control group, patients with CAD showed a higher leukocyte, neutrophil and monocyte counts and lower lymphocyte counts. NLR and MLR were higher in CAD group.

**Table 1:** Baseline characteristics of the study population

Variable	Control Group	CAD Group	*P* value
	(*n* = 162)	(*n* = 381)	
Age (years)	55.41 ± 9.28	62.79 ± 9.52	<0.01
Male, n (%)	57(35%)	211(55%)	< 0.01
Family history, n (%)	20(12%)	55(14%)	0.52
Smoking, n (%)	46(28%)	186(49%)	< 0.01
DM, n (%)	25(15%)	139(36%)	< 0.01
HT, n (%)	85(52%)	291(76%)	< 0.01
HGB (g/L)	135.33 ± 15.87	136.53 ± 15.53	0.42
Platelet (10^9^/L)	221.32 ± 51.31	211.14 ± 53.40	0.04
Leukocyte (10^9^/L)	6.07(4.99–7.13)	6.60(5.60–7.70)	< 0.01
Neutrophil (10^9^/L)	3.42(2.95–4.41)	4.24(3.40–5.10)	< 0.01
Monocyte (10^8^/L)	3.02(2.51–3.62)	3.92(3.22–4.94)	< 0.01
Lymphocyte (10^9^/L)	1.95(1.49–2.29)	1.70(1.36–2.19)	< 0.01
FBG (mmol/L)	5.35(4.93–5.88)	6.22(5.35–7.76)	< 0.01
TC (mmol/L)	4.84 ± 1.10	4.99 ± 1.13	0.15
TG (mmol/L)	1.51(1.06–2.23)	1.51(1.13–2.30)	0.36
LDL (mmol/L)	2.65(2.12–3.25)	2.73(2.23–3.43)	0.29
HDL (mmol/L)	1.16(0.94–1.39)	1.07(0.92–1.30)	0.03
SCr (μmol/L)	71.34(59.27–83.51)	75.96(64.82–89.80)	< 0.01
UA (μmol/L)	334.12 ± 93.41	335.56 ± 91.61	0.87
NLR	1.87(1.42–2.36)	2.47(1.86–3.34)	< 0.01
MLR	0.16(0.13–0.21)	0.23 (0.17–0.30)	< 0.01
Prior Medications			
Aspirin, n (%)	133(82%)	320(84%)	0.59
Beta-blocker, n (%)	61(38%)	170(45%)	0.13
ACEI/ARB, n (%)	70(43%)	194(51%)	0.10
Stain, n (%)	129(80%)	317(83%)	0.32

*ACEI* angiotensin converting enzyme inhibitor, *ARB* angiotensin receptor blocker, *DM* diabetes mellitus, *FBG* fasting blood-glucose, *HDL* high-density lipoprotein, *HGB* Hemoglobin, *HT* hypertension, *LDL* low-density lipoprotein, *MLR* Monocyte to lymphocyte ratio, *NLR* neutrophil to lymphocyte ratio, *SCr* serum creatinine, *TC* total cholesterol, *TG* triglyceride, *UA* uric acid

### MLR is the independent risk factor of the presence of CAD

Multivariate logistic analysis was used to assess 15 clinicopathological characteristics: age, gender, smoking, hypertension, diabetes, fasting blood glucose, HDL, creatinine, leukocyte, neutrophil, monocyte, lymphocyte, platelet, NLR and MLR. Results showed in Table 2 demonstrated that MLR (OR: 3.94, 95% CI: 1.20–12.95) was the independent risk factor of CAD, together with age, male, hypertension, fasting blood glucose and NLR.

**Table 2:** Multivariate logistic regression analysis to assess predictors of CAD

Variable	β	Wald	*P* value	OR	95% CI
Age	0.09	35.29	< 0.01	1.09	1.06–1.12
Male	0.82	4.69	0.03	2.28	1.08–4.79
MLR	1.37	5.11	0.02	3.94	1.20–12.95
NLR	0.81	5.47	0.02	2.24	1.14–4.42–
HT	0.59	5.54	0.02	1.81	1.10–2.97
FBG	0.36	14.66	< 0.01	1.44	1.19–1.73

*CI* confidential interval, *FBG* fasting blood-glucose, *HT* hypertension, *MLR* Monocyte to lymphocyte ratio, *NLR* neutrophil to lymphocyte ratio; *OR* odds ratio

### The efficiency of MLR in detecting CAD

**Fig. 1:**
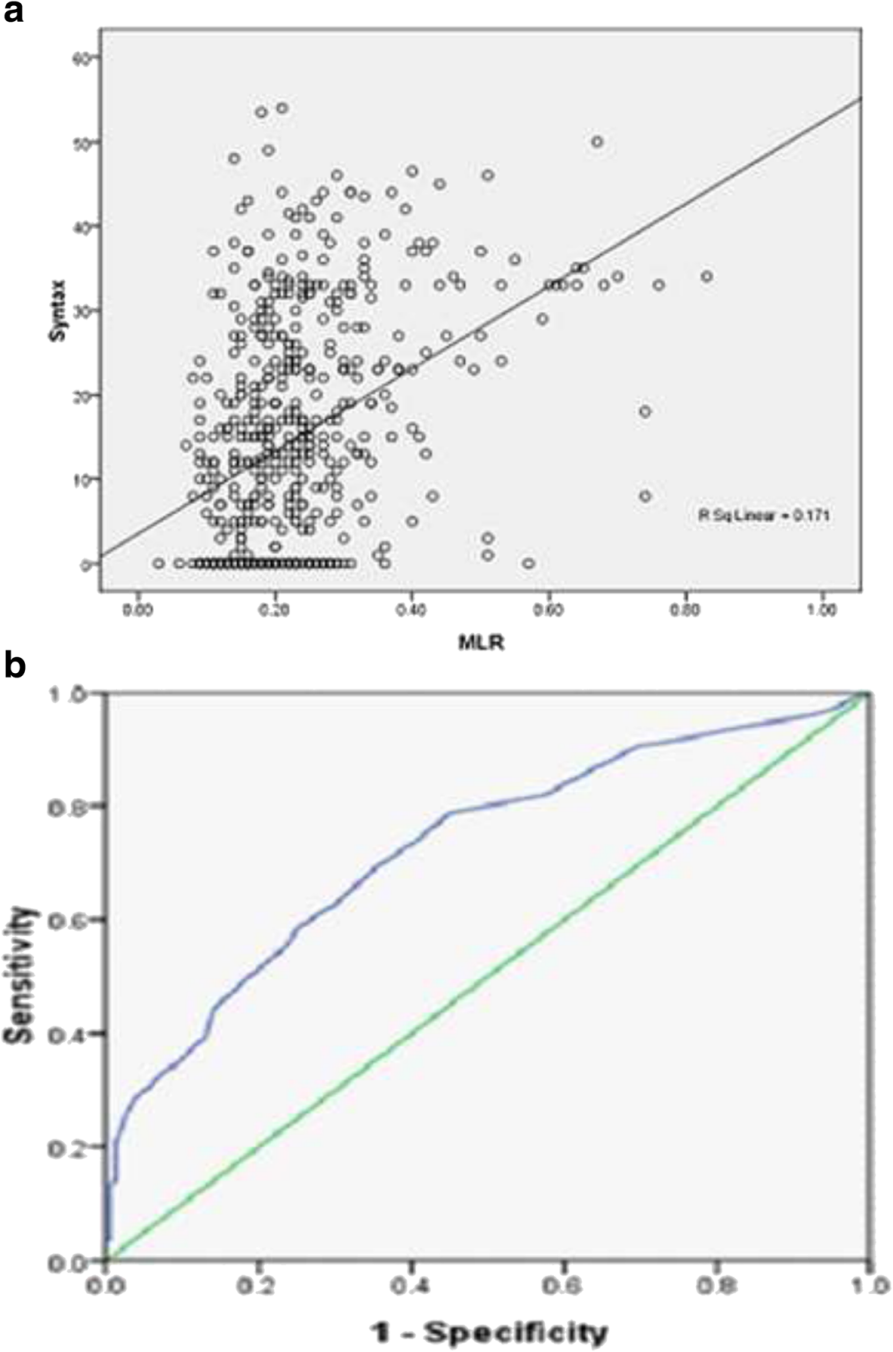
Diagnostic accuracy of circulating MLR in patients with CAD were analyzed by ROC curve; **a** scatter diagram; **b** ROC curve of MLR. MLR: monocyte to lymphocyte ratio; CAD: coronary artery disease

ROC curve analysis was applied to test the efficiency of MLR in detecting CAD with an AUC of 0.727 (95% CI: 0.683–0.771), Fig. 1b. With a cut-off level of 0.18, MLR predicted CAD with a sensitivity of 69.03% and specificity of 64.81%.

### Baseline characteristics of the study population based on coronary atherosclerosis severity

**Fig. 2:**
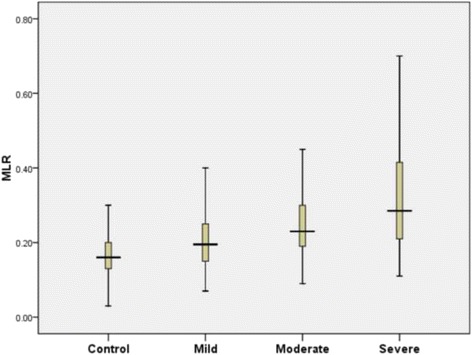
Comparison of MLR values according to the Syntax score. MLR: monocyte to lymphocyte ratio

On the basis of Syntax score to assess coronary atherosclerosis severity, 382 CAD patients were divided into three groups (Syntax score: mild =1–22, moderate 23–32, and severe ≥ 33). The control group consisted of 162 patients with normal coronary arteries, the same as mentioned in Table 1. The distribution of patients’ clinicopathological characteristics were presented in Table 3. Significant differences between severity of coronary atherosclerosis and age, gender, smoking, hypertension, diabetes, fasting blood glucose, creatinine, leukocyte, neutrophil, monocyte, lymphocyte, NLR and MLR were demonstrated. The MLR level in severe atherosclerosis group was statistically higher than that of other three groups (*p* < 0.001, Fig. 2).

**Table 3:** Baseline characteristics of the study population based on coronary atherosclerosis severity

Variable	Control (*n* = 162)	Mild (*n* = 213)	Moderate (*n* = 90)	Severe (*n* = 78)	*P* value
Age(years)	55.41 ± 9.28	61.35 ± 9.20	62.78 ± 9.08	66.76 ± 9.85	< 0.01
Male, n (%)	57(35%)	109(51%)	56(62%)	46(59%)	< 0.01
Family history, n(%)	20(12%)	26(12%)	14(16%)	15(19%)	0.41
Smoking, n (%)	46(28%)	93(44%)	50(56%)	43(55%)	< 0.01
DM, n (%)	25(15%)	60(28%)	45(50%)	34(44%)	< 0.01
HT, n (%)	85(52%)	157(74%)	67(74%)	67(86%)	< 0.01
HGB (g/L)	135.33 ± 15.87	136.52 ± 14.30	138.84 ± 17.09	133.88 ± 16.63	0.18
Platelet (10^9^/L)	221.32 ± 51.31	215.01 ± 53.60	206.66 ± 57.82	205.73 ± 46.94	0.08
Leukocyte (10^9^/L)	6.07(4.99–7.13)	6.41(5.36–7.50)	6.92(6.00–8.18)	6.67(5.80–8.08)	< 0.01
Neutrophil (10^9^/L)	3.42(2.95–4.41)	3.90(3.20–4.63)	4.73(3.81–5.49)	4.67(3.80–5.70)	< 0.01
Monocyte (10^8^/L)	3.02(2.51–3.62)	3.52(3.04–4.38)	4.24(3.36–4.92)	4.81(3.56–5.62)	< 0.01
Lymphocyte (10^9^/L)	1.95(1.49–2.29)	1.84(1.46–2.28)	1.70(1.37–2.06)	1.37(1.10–1.91)	< 0.01
FBG (mmol/L)	5.35(4.93–5.88)	6.04(5.27–7.24)	6.74(5.53–9.41)	6.34(5.56–8.15)	< 0.01
TC (mmol/L)	4.84 ± 1.10	5.03 ± 1.10	4.88 ± 1.19	5.04 ± 1.13	0.35
TG (mmol/L)	1.51(1.06–2.23)	1.57(1.17–2.30)	1.47(1.12–2.31)	1.42(1.04–2.19)	0.34
LDL (mmol/L)	2.65(2.12–3.25)	2.76(2.23–3.35)	2.57(2.09–3.44)	2.88(2.29–3.48)	0.40
HDL (mmol/L)	1.16(0.94–1.39)	1.09(0.92–1.29)	1.04(0.92–1.27)	1.15(0.90–1.31)	0.12
SCr (μmol/L)	71.34(59.27–83.51)	73.36(62.94–86.25)	79.35(65.35–92.30)	81.64(65.85–92.96)	< 0.01
UA (μmol/L)	334.12 ± 93.41	331.93 ± 88.51	335.62 ± 88.79	345.44 ± 102.95	0.74
NLR	1.87(1.42–2.36)	2.09(1.64–2.77)	2.73(2.24–3.34)	3.37(2.50–4.75)	< 0.01
MLR	0.16(0.13–0.21)	0.20(0.15–0.25)	0.23(0.19–0.31)	0.29(0.21–0.43)	< 0.01

*DM* diabetes mellitus, *FBG* fasting blood-glucose, *HDL* high-density lipoprotein, *HGB* Hemoglobin, *HT* hypertension, *LDL* low-density lipoprotein, *MLR* Monocyte to lymphocyte ratio, *NLR* neutrophil to lymphocyte ratio, *SCr* serum creatinine, *TC* total cholesterol, *TG* triglyceride, *UA* uric acid

### MLR is the independent predictor of the lesion severity in CAD

An ordinal multivariate logistic regression was carried out to investigate which factors could be favorable for predicting the severity of the lesion.. The regression result in Table 4 demonstrated that age, smoking, diabetes, hypertension, fasting blood glucose and MLR were independent predictors for the severity of coronary lesion. MLR was an independent predictor of the coronary lesion severity (OR: 2.05, 95%CI: 1.15–3.66), while NLR was not. In the correlation analysis, MLR has significant association with the Syntax score (*r* = 0.437, *p* < 0.001, Fig. 1a).

**Table 4:** Result of ordinal logistic regression analysis

Variable	β	Wald	*P* value	OR	95% CI
Age	0.06	41.03	< 0.001	1.06	1.04–1.08
Smoke	0.67	8.3	0.004	1.95	1.24–3.08
DM	0.43	4.06	0.044	1.54	1.01–2.36
HT	0.61	10.18	0.001	1.85	1.27–2.69
FBG	0.14	9.61	0.002	1.15	1.05–1.25
MLR	0.72	5.93	0.015	2.05	1.15–3.66

*DM* diabetes mellitus, *FBG* fasting blood-glucose, *HT* hypertension, *MLR* monocyte to lymphocyte ratio

### The diagnostic efficiency of MLR in detecting the severe coronary lesion

**Fig. 3:**
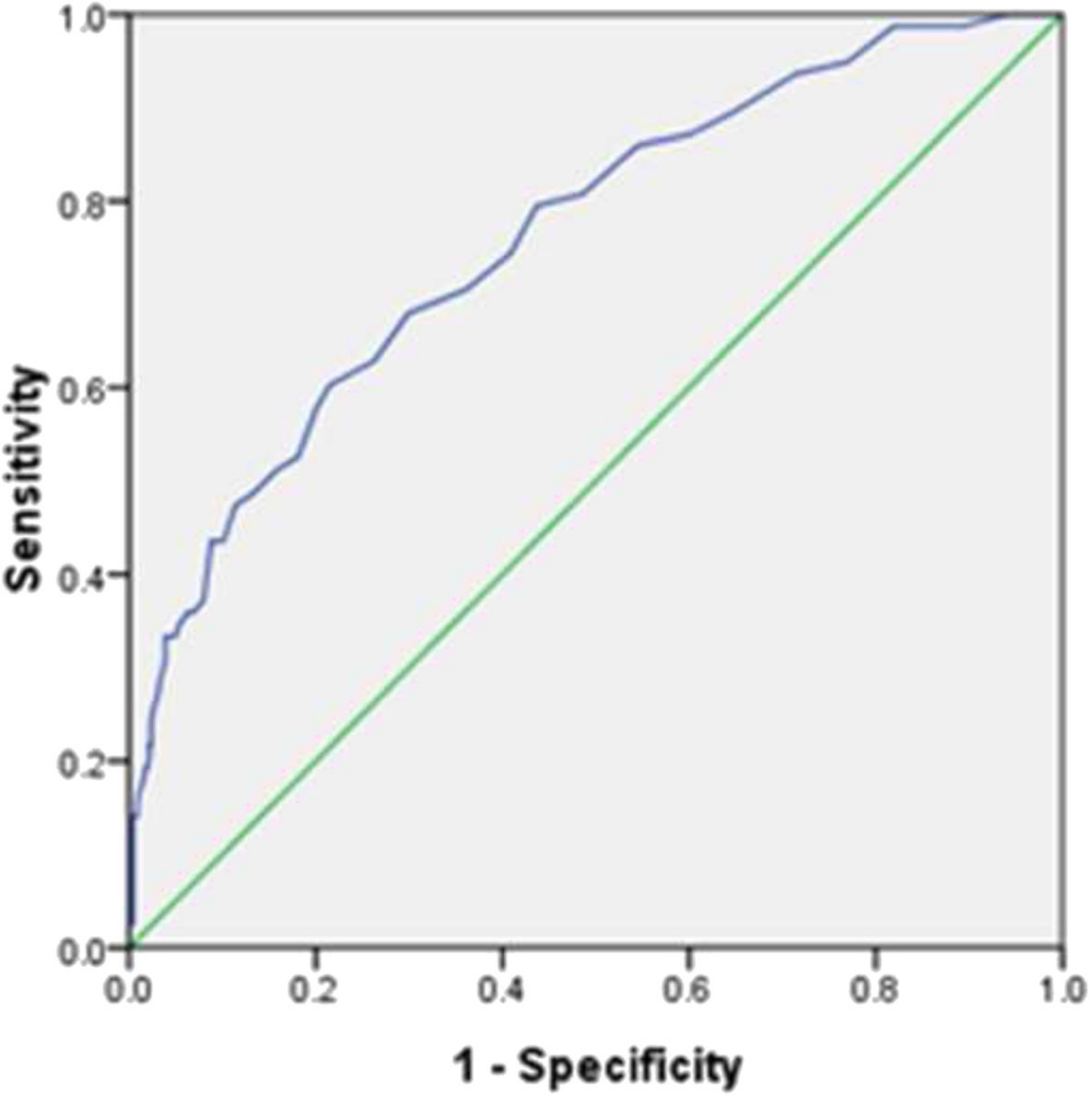
ROC curve for the Diagnostic accuracy of MLR in severe atherosclerosis. MLR: monocyte to lymphocyte ratio

ROC curve was used to analysis the efficiency of MLR in detecting the severe coronary lesion based on Syntax score. A cut-off point of 0.25 for MLR predicted severe coronary lesion with a sensitivity of 60.26% and specificity of 78.49% (ROC area under curve: 0.761, 95% CI: 0.702–0.820, *p* < 0.001, Fig. 3).

## Discussion

MLR calculated as a simple ratio between monocyte and lymphocyte, has been evaluated as an inflammatory response biomarker in various cancers and tuberculosis [21–24], while there is still no report on the relationship between MLR and coronary lesion severity. To the best of our knowledge, our study is the first report to investigate whether there is an association of MLR with coronary lesion severity in CAD. In total, 543 patients were enrolled in our retrospective study, MLR (OR: 3.94, 95%CI: 1.20–12.95) together with NLR, age, male, hypertension, fasting blood glucose were proved to be independent risk factors of CAD. In this study, we also demonstrated that circulating MLR level was a predictor of coronary lesion severity (OR: 2.05, 95%CI: 1.15–3.66), with better performance compared with NLR in terms of logistic analysis. Besides, there is no significant relationship observed between NLR and lesion severity in CAD patients, which is different from previous studies [18, 20].

Atherosclerosis is characterized as a chronic and lasting inflammatory process of arteries, like other inflammatory diseases, characterized by infiltration of immune cells, including monocyte, neutrophils and lymphocyte [10]. The pathogenesis and progression of atherosclerosis lesions is a complex process in which multi-inflammatory factors play a central role [26, 27]. Accumulations of monocytes and monocyte-derived phagocytes are remarkable in the arterial wall, contributing to chronic inflammation process and the formation, exacerbation and complications of atherosclerosis. Monocytes can recruit to the artery wall, differentiate into macrophages and activate the production of proinflammatory cytokines secretion, matrix metalloproteinases, and reactive oxidative species which play a key role in the initiation and formation, or rupture of atherosclerotic plaque [28]. And monocytes phenotype modulation has become a specific therapeutic target for the prevention and treatment of cardiovascular diseases [29]. The lymphocyte represents a potentially important immune cell in cardiovascular disease. Lymphocytopenia, because of increased lymphocytes apoptosis, is a common hall mark of chronic inflammatory status, which is thought to be a negative index of anti-inflammation, post-infarct cardiac healing and remodeling [30, 31]. Nunez et al reported that low lymphocyte count was associated with the increased risk of myocardial infarction or death through the analysis in 1030 patients [32]. In addition, low lymphocyte was found to be reflective of impaired coronary microcirculation which had been validated as a relevant pathogenetic mechanism for CAD [33, 34]. High monocytes and low lymphocytes were confirmed to be independent risk indicators of cardiovascular diseases [16]. Therefore, MLR integrating the risk of these two subtypes index into a single risk factor may be a better risk factor of coronary lesion severity. In our study, increased monocyte and lower lymphocyte were found in CAD patients, which led to elevated MLR level. Moreover, our study suggested that an MLR > 0.18 predicted CAD with a sensitivity of 69.03% and specificity of 64.81%.

Syntax score has been applied to evaluate the risk stratification of CAD. Several Trials have demonstrated that patients with a relatively high Syntax score have worse cardiovascular outcomes [6–8]. In our study, MLR level was positively related to the Syntax score (*r* = 0.437, *p* < 0.001), which reflected that patients with relatively higher MLR might have more severe coronary stenosis and MLR could be helpful in predicting the severity of the lesion. Based on ROC curve analysis, MLR > 0.25 predicted severe lesion with a sensitivity of 78.49% and specificity of 60.26%, which supported MLR could be used to identify severe lesion.

In conclusion, we consider that MLR, a widely available, inexpensive, and robust inflammatory biomarker, could be helpful to predict CAD and evaluate the severity of coronary lesion.

### Study limitations

The major limitation of our present research is the limited study population, and a single center study, other than multiple centers and cross-sectional research. Besides, coronary angiography was the only means to evaluate coronary lesion. Some limitations existing in angiography include the inherently limited ability to identify only surface morphology and its inability to subsurface morphology and internal plaque composition. Other inflammatory markers such as C reactive protein, interleukin-6 and tumor necrosis factor-alpha, were not evaluated in the study. Thus, further efforts on intravascular ultrasound and optical coherence tomography may provide more useful suggestions on the evaluation of coronary lesion.

## Conclusions

To our best knowledge, our study is the first report on the relationship between MLR and coronary lesion severity. We found that MLR was a risk factor of atherosclerosis, and can be a predictor for the severity of the lesion rather than NLR. Also MLR was significantly correlated with Syntax score. Therefore we consider that patients with CAD who have a higher MLR also have more atherosclerosis involvement and we also suggest a preprocedural MLR, a widely available, inexpensive, and robust inflammatory biomarker, could be helpful for cardiac risk stratification.

## Abbreviations

CAD: Coronary artery disease
FBG: Fasting blood-glucose
HDL: High density lipoprotein cholesterol
HGB: Hemoglobin
LDL: Low density lipoprotein cholesterol
MLR: Monocyte to lymphocyte ratio
NLR: Neutrophil to lymphocyte ratio
ROC: Receiver-operating characteristic
SCr: Serum creatinine
TC: Total cholesterol
TG: Triglycerides
UA: Uric acid
